# Antibiotic Susceptibility of *Staphylococcus* Species Isolated in Raw Chicken Meat from Retail Stores

**DOI:** 10.3390/antibiotics10080904

**Published:** 2021-07-23

**Authors:** Erinda Lika, Nikola Puvača, Dejan Jeremić, Slobodan Stanojević, Tana Shtylla Kika, Sonila Cocoli, Rosa de Llanos Frutos

**Affiliations:** 1 Faculty of Veterinary Medicine, Agricultural University of Tirana, Koder Kamez, 1029 Tirana, Albania; elika@ubt.edu.al (E.L.); tana.shtylla@ubt.edu.al (T.S.K.); scocoli@ubt.edu.al (S.C.); 2 Faculty of Health, Jaume I University, Avinguda de Vicent Sos Baynat, s/n, 12071 Castelló de la Plana, Spain; dellanos@uji.es; 3 Department of Engineering Management in Biotechnology, Faculty of Economics and Engineering Management in Novi Sad, University Business Academy in Novi Sad, Cvećarska 2, 21000 Novi Sad, Serbia; 4 Faculty of Business and Financial Studies, University of Business Studies in Banja Luka, Jovana Dučića 23a, 78000 Banja Luka, Bosnia and Herzegovina; djeremic@sequestergroup.com; 5 Faculty of Applied Management, Economics and Finance, University Business Academy in Novi Sad, Jevrejska 24/1, 11000 Belgrade, Serbia; slobodan.stanojevic@mef.edu.rs

**Keywords:** antibiotic resistance, microbes, raw meat, foodborne pathogens, retail stores, *S. aureus*

## Abstract

The study was aimed at evaluating the presence of antibiotic-resistant *Staphylococcus aureus* in retailed raw chicken meat from retail stores intended for human consumption. The presence, characterization, and antibiotic susceptibility of *S. aureus* from 38 retail raw chicken meat samples was performed using a standard microbiological method involving mannitol salt agar (MSA) and Mueller-Hinton agar (MHA). All the samples were positive for *Staphylococcus* species, of which 34 (89.5%) were positive for *S. aureus*. The *S. aureus* isolates were most resistant to tetracycline (88.24%), erythromycin (82.35%), and chloramphenicol (61.77%). Nevertheless, decreased resistance towards gentamycin (23.53%) and cotrimoxazole (38.24%) were recorded. All the *S. aureus* isolates in this study were resistant to cloxacillin, amoxicillin, and augmentin (amoxicillin + clavulanic acid). The present findings show how the raw chicken meat samples could be a potential source of multidrug-resistant *S. aureus* strains dissemination. Therefore, this study suggests high-level contamination of meat with multidrug-resistant *S. aureus* and highlights the public health consequences of consuming such products. Undoubtedly, uncontrolled drugs in food animal production as growth stimulators or medicinal treatment present a possible consequence to people’s health. Having the aforementioned in mind, there is a necessity to control the use of drugs and monitor any residues left in the food intended for human consumption.

## 1. Introduction

Meat and meat products are among the most consumed foods and are important sources of all the B-complex vitamins, as well as minerals, proteins, and amino acids in humans.

Meat of animal origin is the primary source of protein and valuable qualities of vitamins for most people in many parts of the world, thus it is essential for the growth, repair, and maintenance of body cells and necessary for our everyday activities [1,2]. Meat is the main source of iron in heme form, which is one of the most deficient micronutrients in humans [3]. Due to the chemical composition and biological characteristics, meats are highly perishable foods providing an excellent source of nutrients for the growth of several hazardous microorganisms that can cause infection in humans, resulting in spoilage of the meat and, therefore, economic loss [4,5]. The microbial pathogens found in meat microorganisms are *Listeria monocytogenes* [6], *Micrococcus* spp. [7], *Staphylococcus* spp. [8], *Clostridium* spp. [9], *Bacillus* spp. [10], *Brochotrix thermophacta* [11], *Salmonella* spp. [12], *Escherichia coli* [13], *Serratia* spp. [14] and *Pseudomonas* spp. [15]. Growth of foodborne pathogens such as *Salmonella*, and toxin-producing strains of *E. coli*, *L. monocytogenes*, *C. perfringens,* and *S. aureus* are the main concern with meat and poultry products [16,17,18]. These bacteria are the most common cause of foodborne illnesses. Besides poultry meat, *S. aureus* as well as Methicillin-resistant *S. aureus* can be found in swine [19] and cattle [20] meat.

The most significant Gram-positive organism that has gained attention because of its associated hospital- and community-acquired infections is *S. aureus* [21,22,23]. This bacterium multiplies quickly at room temperature to produce toxins that cause food poisoning [24]. Naturally, its distribution is very common globally, but the most important infection origin of *S. aureus* is food [25]. According to Scallan et al. [26], *S. aureus* has come into the spotlight as a foodborne pathogen with more than 200,000 estimated yearly infections domestically acquired within the US. The number of cases may actually be higher than this, however the lower known incidences of *S. aureus* foodborne disease could be due to misdiagnosis, inadequate sample collection and laboratory analyses, lack of seeking medical health care by the affected persons (complicating the laboratory confirmation), and lack of routine surveillance of clinical stool specimens for *S. aureus* [27].

Staphylococcal food contamination represents the greatest economically significant foodborne illness [28] and produces gastrointestinal illness through a wide variety of toxins [29], including staphylococcal enterotoxins characterized by vomiting and diarrhea within 2 to 6 h after the consumption of contaminated food [30,31,32]. A large number of daily consumed foods serve as an optimum growth medium for *S. aureus* [27], and this varies from country to country, especially due to different habits in food consumption [33]. *S. aureus* and other pathogens in meat result from improper hygienic practices at the point of handling by slaughter personnel during meat processing, and other faulty abattoir processes such as improper evisceration of animals which increases the chances of cross-contamination of gut pathogens to meat [34,35].

Residues from medicines, insecticides, herbicides, and other compounds used in daily agricultural practice could be detected in minor quantities in food of animal origin. A few hundred compounds, mainly antibiotics, have been used to cure animals and protect their health, however, some of them have also been used to enhance food animal production. Among many unethically used compounds, the most often used are antimicrobials, β-adrenoreceptor blocking agents, ivermectin, sedatives, coccidiostats, vasodilatory drugs, and painkillers. Residues from such compounds in food products are a major public health concern, especially with rising interest and increased awareness of the potential deposits of drugs and their metabolites in the meat and meat products consumed by humans, as well as the development of antimicrobial resistance (AMR). Treatment of *S. aureus* infections involves the use of antibiotics [36]. However, the use and misuse of antibiotics prophylactically or sub-therapeutically to prevent bacterial infections in livestock and the resultant residue, in general have been responsible for the development of multidrug-resistant bacterial isolates and a significant public health issue. Several microorganisms have developed resistance to various antibiotics, which have triggered the expansion of novel antibiotics with a higher resistance level [37,38,39].

Numerous studies have shown the presence of *S. aureus* in raw meat and meat products from retail stores with a prevalence below 1% in Asia [40], up to around 12% in Europe [41].

The study was aimed at evaluating the antimicrobial resistance profile of *S. aureus* isolates in retail raw chicken meat intended for human consumption.

## 2. Results and Discussion

We examined a total of 38 samples of raw chicken meat from retail stores for the presence of *Staphylococcus* spp. Our results showed that all 38 samples were positive for *Staphylococcus* spp. of which 89.5% (34 samples) of the confirmed isolates were *S. aureus*. All the isolates fermented mannitol salt agar and appeared golden yellow, showing the biochemical characteristics previously reported by Konuku et al. [42] for *Staphylococcus* spp. Our results of the occurrence of *Staphylococcus* spp. in meat samples is in agreement with previously reported results that describe *S. aureus* as a common pathogen of raw meats [41,43].

**Figure 1:**
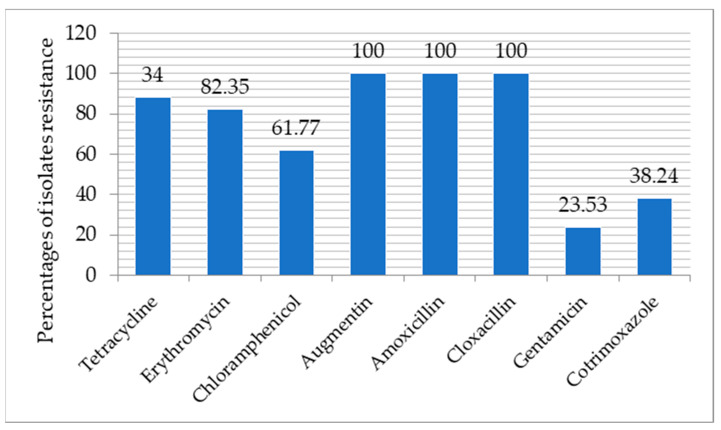
Percentage of *S. aureus* isolates resistant to common antibiotics (number of positive samples; *n* = 34), %.

The presence of antimicrobial-resistant bacteria in meat has been widely reported from different parts of the world. The use of antibiotics in livestock and the resultant residue contribute to high antibiotic resistance levels of *S. aureus* found in meat products. All the *S. aureus* isolates in this study were resistant to cloxacillin, amoxicillin, and augmentin (Figure 1). In accordance with the findings of our research, Waters et al. [44] also reported strains of *S. aureus* in US meat and poultry resistant to ciprofloxacin, quinupristin/dalfopristin, clindamycin, erythromycin, oxacillin, and daptomycin. Varying resistance of *S. aureus* from raw meat has been reported by many authors, ranging from 25.00% to 73.30% [34,43,45,46].

*S. aureus* strains were least resistant to gentamycin (23.53%) and cotrimoxazole (38.24%). Some authors have reported that *S. aureus* gentamicin-resistant isolates from raw meat can range up to 19.40% [34,47,48,49]. This may lower percentage may be because it is in injection form and hardly used, unlike a vast majority of antibiotics that come in capsule or tablet forms. For cotrimoxazole, contrary to the findings of this study, Effah et al. [50] reported a 57.80% resistance of Methicillin-resistant *S. aureus* isolated from raw meat. Other authors, however, reported varying resistances (8.00 to 34.2%) to Methicillin-resistant *S. aureus* (MRSA) from humans [51,52].

*S. aureus* is among the most prevalent cause of clinical infections globally and has garnered substantial public attention due to the increased mortality associated with the multidrug resistance phenomenon. Our findings also show the potential dissemination of multidrug-resistant *S. aureus* strains in the raw chicken meat samples examined. *S. aureus* isolates were multidrug-resistant to at least three antibiotics tested (Table 1). Consistent with the findings of our research, Effah et al. [50] reported multidrug resistance of MRSA to 16 antibiotics, of which 6 of those antibiotics were among those herein tested. The presence of multidrug-resistant strains poses a severe public health risk, as well as other emerging novel diseases [53].

Antibiotic susceptibility patterns for the raw chicken meat associated bacteria identified in this study are presented in Table 2. Antibiotics included in the testing were augmentin, amoxicillin, erythromycin, tetracycline, cloxacillin, gentamicin, cotrimoxazole, and chloramphenicol. According to Kovačević et al. [54], the most used antibiotics in inflammation therapy in food animals are penicillin, streptomycin, gentamicin, tetracycline, cephalexin, sulfonamides, and enrofloxacin.

Correspondence analysis was used to describe the bactericidal potential of different antibiotics on bacteria isolated from the raw chicken meat samples and shows associations of different bacteria and the evaluated antibiotics in terms of bacteria resistance (R) or sensitivity (S). As previously stated, *Staphylococcus* spp. have shown resistance toward all investigated antibiotics, with intermediate (I) resistance towards COT and GEN. As in our findings, Regecová et al. [55] investigated antimicrobial resistance of coagulase-negative Staphylococci isolated from sea fish meat. They observed that all isolates showed antimicrobial resistance to seven antibiotics, with most isolates resistant to ampicillin (AMP) and GEN. Ljubojević et al. [56] pointed out significant problems of widespread use of tetracyclines in poultry farming. Irregular and unprescribed usage of antibiotics may have resulted in the development and transmission of resistant strains from poultry to humans via the food chain. Furthermore, Puvača and de Llanos [57] have explained mechanisms of transmission and resistance via the fecal-oral route between humans, environmental sources, and food and pet animals in their review. The significant impact on drug resistance could also be due to inappropriate antibiotic medical decision therapy [58].

The minimum inhibitory concentrations (MICs) and minimal bactericidal concentrations (MBCs) of *S. aureus* and *Staphylococcus* spp. to augmentin, amoxicillin, and cloxacillin antibiotics are shown in Table 3. Our results show that all isolates of *S. aureus* and *Staphylococcus* spp. found in raw chicken meat samples were multidrug resistant to these three antibiotics. Recorded MIC/BMC concentration in our study regarding *S. aureus* was as follows: AMX > CXC > AUG (8.2/16.4 mg/L > 6.4/12.8 mg/L > 5.8/11.6 mg/L); while *Staphylococcus* spp. recorded a similar trend (7.6/15.4 mg/L > 7.3/14.6 mg/L > 4.6/9.2 mg/L).

In the research of Thorburn et al. [59], post-antibiotic and post-β-lactamase inhibitor effects of amoxicillin were investigated. The effects of AMX were investigated on several bacteria including *S. aureus* and *E. coli* and a necessity for antibiotic dosage reduction was observed. Also, Sader et al. [60] highlighted that the usage of third-generation antibiotics exhibits more balanced spectrums of activity against pathogens and infections when compared with other antibiotics, but only in strictly controlled therapy.

## 3. Materials and Methods

The fresh raw chicken meat samples (thighs, breasts, and wings of the same chicken) were randomly purchased in January 2021, from a total of 38 different retail meat stores originating from different producers in a territory of the Autonomous Province of Vojvodina located in the Republic of Serbia. Chickens came from independent processing plants. Meat samples were packed in a protective atmosphere and transferred in sterile flask coolers at +4 °C, upon which samples were sent to the laboratory for further analysis.

A total of 25 g of mixture of meat samples were ground and aseptically weighed into a stomacher bag containing 225 mL of sterile saline solution. This was followed by homogenization in a stomacher (Lab. Lemco 400, Worthing, West Sussex, UK) for about 100 s. To prepare decimal dilutions, 1.0 mL of the initial suspension (10^−1^) to 9.0 mL of peptone saline diluent (PSD) (to a tolerance of ±2% at ambient temperature), avoiding contact between the pipette tip and the diluent, was transferred and mixed carefully using a vortex mixer (Drawell, Chongqing, China) for 5–10 s. PSD was prepared by suspending 15 g of Peptone Water in 1000 mL of distilled water, followed by the addition of the test carbohydrate until completely dissolved, and then dispensed into inverted Durham’s tubes and sterilized by autoclaving at 121 °C for 15 min. The time lapse between preparation of the initial suspension and the beginning of preparation of the further dilutions did not exceed 30 min, and the overall time lapse between preparation of the initial suspension and inoculation of the plating media did not exceed 45 min. After a ten-fold serial dilution, 0.1 mL of diluted homogenate was spread-plated in duplicates on mannitol salt agar (MSA) supplemented with egg yolk-tellurite emulsion (Oxoid Limited, Basingstoke, Hampshire, UK), and incubated at 35 °C for 24 h.

From each plate, typical colonies of *Staphylococcus* spp. with similar morphologies were isolated and cultured separately on MSA before storing in Nutrient Agar Slant for confirmation. Identification of bacterial isolates was confirmed using the Cowan and Steel [61] manual for the Identification of Medical Bacteria, and Bergey and Holt [62] manual of Determinative Bacteriology.

Antibiotic sensitivity patterns of all the confirmed *Staphylococcus* spp. were performed using the standard disk diffusion method on Mueller-Hinton agar (Titan, Biotech Ltd.) following the procedures recommended by the Clinical and Laboratory Standards Institute (CLSI) [63]. Ten commonly used antibiotics (µg/disc) such as augmentin (30 µg) (amoxicillin + clavulanic acid), amoxycillin (25 µg), erythromycin (5 µg), tetracycline (10 µg), cloxacillin (5 µg), gentamycin (10 µg), cotrimoxazole (25 µg), chloramphenicol (30 µg) were tested. From an overnight culture in Brain Heart Infusion Broth, a 10^8^ cell/mL (0.5 MacFarland turbidity standards) bacterial culture was prepared in sterile saline solution, from which 0.1 mL was inoculated onto Mueller-Hinton agar, after which antibiotic discs were carefully and aseptically placed on the surface of the agar. The plates were incubated at 37 °C for 24 h. Inhibition zones for various isolates were measured and interpreted as sensitive, intermediate, or resistant according to the CLSI [64,65]. When a single isolate was resistant to one key antimicrobial agent, multidrug resistance was registered [66].

The Brain Heart Infusion (BHI) Broth microdilution method was used to establish the minimal inhibitory concentrations (MIC) corresponding to the Clinical and Laboratory Standards Institute guideline [67]. The 180 µL aliquots of Tryptone soya broth were added to 96-well microtiter plates. As the final step, 20 µL of the standardized bacterial suspension (10^8^ cell/mL) was inoculated into each well. The assay was performed in a total volume of 200 µL with final antimicrobial concentrations ranging from 100 to 0.09 mg/L, while the final microbial concentration was 10^5^ CFU/mL. Plates were incubated at 37 °C, during 6 h in darkness. After visual examination, the plates were additionally incubated for 18 h. Change of color from blue (oxidized) to pink (reduced) indicated the growth of bacteria. MIC was defined as the lowest concentration at which the color change occurred [68]. Bacterial growth was determined by measuring absorbance at 600 nm.

To determine the minimum bactericidal concentration (MBC), known as the lowest concentration that reduces the bacterial population 99.9% after incubation at 35 °C for 24 h, 100 μL of the microtiter wells with no visible growth in the MIC determination assay was transferred to count agar plates (Lab M, International Diagnostics Group Plc, Bury, Lancashire, UK), which were incubated at 37 °C for 24 h. Those wells that yielded plates with no visible colonies were considered to be the MBC.

## 4. Conclusions

The role of food in the spread of pathogens cannot be over-emphasized in public health. Based on our results, raw chicken meat from retail stores remains a potential source in transmitting pathogenic foodborne bacteria. All the samples were positive for *Staphylococcus* species, of which 34 (89.5%) were positive for *S. aureus*. The *S. aureus* isolates were most resistant to tetracycline (88.24%), erythromycin (82.35%), and chloramphenicol (61.77%), while decreased resistance toward gentamycin (23.53%) and cotrimoxazole (38.24%) was recorded. All the *S. aureus* isolates in this study were resistant to cloxacillin, amoxicillin, and augmentin (amoxicillin + clavulanic acid). Therefore, there is the need for adequate food processing, especially at a suitable temperature, to reduce the possible microbial contamination in the food products, as well as surveillance of and good hygiene practice by meat handlers in the face of an increasing threat of multidrug-resistant *S. aureus* both in animals and humans. From our findings, it was determined that raw chicken meat from retail stores can be classified as “very high additional risk” or even as “high additional risk”. This highlights the importance of continued surveillance and the need to take measures in the primary sector to minimize the risk for the consumer.

## Figures and Tables

**Table 1:** Multidrug resistance of *S. aureus* isolates in raw chicken meat samples.

Antibiotics	Isolate’s Resistance (n)
AUG-AMX-CXC-TET-ERY	8
AUG-AMX-CXC-TET-ERY-CHL	6
AUG-AMX-CXC-TET-ERY-CHL-COT	8
AUG-AMX-CXC-TET-ERY-CHL-COT-GEN	6

AUG—augmentin; AMX—amoxicillin; ERY—erythromycin; TET—tetracycline; CXC—cloxacillin; GEN—gentamicin; COT—cotrimoxazole; CHL—chloramphenicol; n—number of isolates.

**Table 2:** Antibiotic susceptibility patterns for the raw chicken meat associated bacteria.

Bacteria	AUG	AMX	ERY	TET	CXC	GEN	COT	CHL
*Staphylococcus* spp.	R	R	R	R	R	R	I	R

AUG—augmentin; AMX—amoxicillin; ERY—erythromycin; TET—tetracycline; CXC—cloxacillin; GEN—gentamicin; COT—cotrimoxazole; CHL—chloramphenicol; I—intermediate; R—resistant.

**Table 3:** Minimum inhibitory concentrations (MICs) and minimal bactericidal concentrations (MBCs) of *S. aureus* and *Staphylococcus* spp. to augmentin, amoxicillin, and cloxacillin antibiotics.

Sample	AUG			AMX			CXC		
	MIC, mg/L	MBC, mg/L	Cutoff, mg/L	MIC, mg/L	MBC, mg/L	Cutoff, mg/L	MIC, mg/L	MBC, mg/L	Cutoff, mg/L
*S. aureus*	5.8	11.6	1.45	8.2	16.4	2.05	6.4	12.8	1.6
*Staphylococcus* spp.	4.6	9.2	1.15	7.6	15.4	1.9	7.3	14.6	1.83

AUG—augmentin; AMX—amoxicillin; CXC—cloxacillin.

## Data Availability

Data is contained within the article.
